# Modern morphological engineering techniques for improving productivity of filamentous fungi in submerged cultures

**DOI:** 10.1007/s11274-016-2148-7

**Published:** 2016-10-07

**Authors:** Anna Antecka, Marcin Bizukojc, Stanislaw Ledakowicz

**Affiliations:** Department of Bioprocess Engineering, Lodz University of Technology, ul. Wolczanska 213, 90-924 Lodz, Poland

**Keywords:** Biomass, Filamentous fungi, Medium osmolality, Microparticle-enhanced cultivation, Productivity

## Abstract

Morphological engineering techniques have recently gained popularity as they are used for increasing the productivity of a variety of metabolites and enzymes in fungi growing in submerged cultures. Their action is mainly associated with the changes they evoke in fungal morphology. Traditional morphological engineering approaches include manipulation of spore concentration, pH-shifting and mechanical stress exerted by stirring and aeration. As the traditional methods proved to be insufficient, modern techniques such as changes of medium osmolality or addition of mineral microparticles to the media (microparticle-enhanced cultivation, MPEC) were proposed. Despite the fact that this area of knowledge is still being developed, there are a fair amount of scientific articles concerning the cultivations of filamentous fungi with the use of these techniques. It was described that in *Ascomycetes* fungi both MPEC or change of osmolality successfully led to the change of mycelial morphology, which appeared to be favorable for increased productivity of secondary metabolites and enzymes. There are also limited but very promising reports involving the successful application of MPEC with *Basidiomycetes* species. Despite the fact that the mineral microparticles behave differently for various microorganisms, being strain and particle specific, the low cost of its application is a great benefit. This paper reviews the application of the modern morphology engineering techniques. The authors critically assess the advantages, shortcomings, and future prospects of their application in the cultivation of fungi.

## Introduction

Filamentous fungi are metabolically versatile microorganisms with a very wide distribution in nature (Archer et al. 2008). They secrete a variety of hydrolytic enzymes that degrade waste organic materials, and many of them are regarded as primary decomposers in nature. They are also able to produce such secondary metabolites as polyketides, alkaloids or peptides. Therefore, an increasing range of fungal species are exploited commercially as sources of enzymes and metabolites in a broad range of industrial processes from food to pharmaceutical applications (Ghaffari-Moghaddam et al. 2014; Marinelli and Marcone 2011). Moreover, the recent availability of fungal genomes has provided a major opportunity to explore and further exploit fungi as a source of enzymes and metabolites. Due to their highly efficient secretion of proteins filamentous fungi are one of the most important cell factories in the production of industrial enzymes. In an industrial context, fungal productivity depends strongly on morphology, although not always directly (Cox et al. 1998).

Filamentous microorganisms present a diverse and complicated range of gross and internal morphologies, which to date remain one of the most intriguing subjects in this field of study. Typically, they consist of hyphae, which are relatively long compared to their width, often branched, and forming extended structures called mycelia (Cox et al. 1998). There are generally two morphological forms of filamentous fungi growth: dispersed morphology and spherical hyphal aggregates, referred to as macroscopic pellets. This diversity of specific morphological forms of filamentous fungi, ranging from the dense spherical pellets to viscous mycelial suspensions, makes high demands on control in submerged cultivations. It is strongly dependent on culture conditions and strictly correlates with the optimal biosynthesis of the desired products. Furthermore, the existence of various morphological forms in the submerged cultures is connected with several engineering issues that have to be solved. In the macroscopic spherical pellets which can reach a diameter of several millimeters limitations in diffusive mass transfer occur, lowering nutrients and oxygen levels, especially in the centers of pellets. On the other hand, the mycelial suspension (dispersed morphology) often decreases mixing efficiency as the viscosity increases to such an extent that it makes stirring difficult. In addition, the convective oxygen transport due to strongly non-Newtonian rheological properties of the cultivation broth is limited; and it is often not clear which morphological form is favorable for the efficient production of metabolites or enzymes.

Fungal morphology can be controlled by means of genetic modifications (McIntyre et al. 2001) and other techniques that utilize the influence of cultivation conditions on fungal morphology. All the actions aimed at controlling fungal morphology are referred to as morphological engineering. According to McIntyre et al. (2001) the term is defined as “tailoring morphologies for specific bioprocesses”.

In order to control fungal morphology at the process level, such traditional methods as varying spore concentration, changing pH level, inducing mechanical stress due to stirring and aeration, changing cultivation temperature and medium composition are implemented (Nielsen et al. 1995; Papagianni 2004; Bizukojc and Ledakowicz 2010). But in many cases they are insufficient, and thus other methods were proposed. These “new approaches” include the addition of microparticles and variation of medium osmolality (Krull et al. 2013).

Various mechanisms of pellet formation have been observed in filamentous microorganisms. One may distinguish spore agglomeration typical for *Aspergilli*, non-agglomerative pellet formation in *Zygomycetes* and *Streptomycetes* and mycelial agglomeration in *Penicilli.* In most cases the actions undertaken by morphological engineering techniques are to interfere with agglomerate (pellet) formation and produce smaller, less dense pellets or even dispersed mycelium. Such conditions often lead to better consumption of substrates and increase of fungal productivity (Kaup et al. 2008; Driouch et al. 2010a; Gonciarz and Bizukojc 2014).

There are many original papers concerning morphological engineering techniques but there are not too many reviews on this topic. As the latest review by Krull et al. was published in 2013, and this area of research develops rapidly, there is a need to review the latest findings. Therefore, the aim of this review is to present the applications of modern morphological engineering techniques in the cultivation of filamentous fungi.

## Microparticle-enhanced cultivation of filamentous fungi

### Microorganisms: Which filamentous fungi?

As the one of the most commonly used fungi are those of the genus *Aspergillus*, therefore the study concerning microparticle-enhanced cultivation (MPEC) methods were mostly concentrated on this genus. Filamentous fungi belonging to the genus *Aspergillus* are claimed to be one of the most intriguing and often uncontrollable organisms because of their complex morphology (Wucherpfennig et al. 2011). With regard to MPEC they were deeply studied by Driouch et al. (2010a, b, 2012). They used two recombinant strains: an uridine auxotropic, α-glucoamylase-producing strain *Aspergillus niger* AB 1.13 and *A. niger* SKAn 1015 with β-fructofuranosidase gene (Driouch et al. 2010a). First, they studied the effect of talc and aluminum oxide microparticles on the formation of the aforementioned enzymes. Next, they made bioprocess optimization of β-fructofuranosidase production with the addition of talc microparticles in a 3-l stirred tank bioreactor operating in the fed-batch mode (Driouch et al. 2010b). Ultimately, they used titanium silicate oxide microparticles in the cultivation of these two fungal species (Driouch et al. 2012).

Gonciarz and Bizukojc (2014) and Gonciarz et al. (2016) studied lovastatin-producing *Aspergillus terreus* ATCC 20542 and cultivated it in shake flasks with talc microparticles (Gonciarz and Bizukojc 2014), and in batch and continuous fed-batch stirred-tank bioreactors (Gonciarz et al. 2016). Similarly, Coban et al. (2015a, b), who studied the enhancement of phytase production by *Aspergillus ficuum* NRRL 3135 with addition of talc or aluminum oxide, started with shake flasks and then continued in fed-batch and continuous bioreactors. This team later changed their interests to lactic acid production by *Rhizopus oryzae* NRRL 395 (Coban and Demirci 2016). In the meantime, Etschmann et al. (2015) studied two fungal strains: *A. niger* DSM 821, being a 2-phenylethanol producer and *Trichoderma atroviridae* IMI 206040 a 6-pentyl-α-pyrone producer. Quite recently *Aspergillus sojae* AsT1, was studied by Yatmaz et al. (2016) to enhance β-mannanase production by addition of talc and aluminum oxide in the shake flask scale.

Of other fungi studied, there were three strains which do not produce asexual spores, but their morphology was also changed after addition of microparticles to the cultivation medium. *Caldariomyces fumago* DSM 1256, a chloroperoxidase producer, was the first species with which MPEC method was used (Kaup et al. 2008). Finally, two basidiomycetes strains *Cerrena unicolor* (Bull. Ex Fr.) Murr. 137 and *Pleurotus sapidus* DSM 8266 (Antecka et al. 2016) were studied to enhance laccase production by employment of MPEC.

Besides the aforementioned most profound studies, simple screening experiments were made with six other fungal species: *Penicillium digitatum*, *Penicillium chrysogenum*, *Emericella nidulans*, *Acremonium chrysogenum*, *Rhizopus oryzae*, *Chaetomium globosum* (Kaup et al. 2008).

### Microparticles: Which ones and how much?

MPEC was conceptualized following a publication by Kaup et al. (2008) in which their results showed a 10-fold increase of chloroperoxidase activity in the culture medium of *C. fumago* DSM 1256 with talc microparticles added. But it did not indicate that all microparticles act in the same way. In the experiments, Kaup et al. (2008) thoroughly examined nine different concentrations (ranging from 0.05 to 25 g l^−1^) of aluminum oxide and talc powder with various particle size distribution. They obtained positive results, but not in each case. The addition of microparticles at concentrations of 15 g l^−1^ for aluminum oxide and 10 g l^−1^ for talc powder led to the increase of biomass formation and chloroperoxidase activity, whereas lower microparticle concentrations stimulated biomass formation to a lesser degree and the higher ones did not significantly enhance biomass and product formation. Additionally, it was proven that only microparticles of a diameter smaller than 42 µm could change *C. fumago* DSM 1256 morphology from pellet to dispersed. Particles of 500 µm diameter did not exert any effect on growth, morphology or production of the enzyme by the fungus. Generally, microparticles of a diameter comparable to the size of spores or spore agglomerates are the most efficient.

Further investigations showed that talc microparticles have been the most often applied. The addition of these microparticles was also studied by Driouch et al. (2010a, b), Gonciarz and Bizukojc (2014), Etschmann et al. (2015) and Coban et al. (2015a). Their results confirmed that the optimal concentration of talc microparticles is about 10–20 g l^−1^. However, it has recently been observed that talc powder is not completely inert in the medium as it releases such amounts of magnesium ions into culture media that they can be physiologically significant. The effect of biomass and productivity enhancement could be partially connected with magnesium ions (Etschmann et al. 2015). The second most popular microparticle is aluminum oxide, which proved to be completely insoluble in water and inactive (Etschmann et al. 2015). Results of other authors confirmed that the most effective aluminum oxide concentration is similar to that of talc, approximately 15–20 g l^−1^ (Driouch et al. 2010a, b; Etschmann et al. 2015; Coban et al. 2015a; Antecka et al. 2016).

Driouch et al. (2012) also applied titanium silicate oxide (8 µm) to stimulate α-glucoamylase by *A. niger* AB 1.13 and β-fructofuranosidase formation by *A. niger* SKAn 1015 and tested microparticle concentrations in the range from 5 to 50 g l^−1^. In this case, 25 g l^−1^ of titanate was an optimum dose. Finally, Etschmann et al. (2015) screened 16 various microparticles of different chemical composition and nominal particle diameter ranging from 5 to 250 µm. Their conclusions were partially unsatisfying, concluding that there is no real rule for MPEC and all the effects are strain-specific, so individual study for each case is required.

### Effects of MPEC: What are the targets?

#### Change of mycelial morphology

In most cases the microparticles in the culture media influence both biomass concentration and fungal morphology, namely the size, shape, and the structure of the mycelia. For example, *C. fumago* DSM 1256 normally grows as densely packed pellets with diameters up to 4 mm. But in the medium containing microparticles of talc or aluminum oxide the mycelium was loosely packed and attained diameters of 0.1–0.5 mm along with a significant amount of single hyphae (Kaup et al. 2008). Similarly, *A. niger* SKAn 1015 forms mycelial clumps of average diameter equal to 1.7 mm, whereas with the addition of talc or aluminum oxide the size of pellets decreased to 0.1 mm, becoming free mycelium with increasing concentrations of microparticles (Driouch et al. 2010a). Visually homogeneous cultures without agglomerates were also achieved by Etschmann et al. (2015) for *A. niger* DSM 821 cultivations, in which 16 various microparticles were used and all of them led to the same dispersed morphological form. In the case of *T. atroviride* IMI 206040 the same studies resulted in pellets of various sizes but no homogeneous cultures were obtained (Etschmann et al. 2015).

**Fig. 1:**
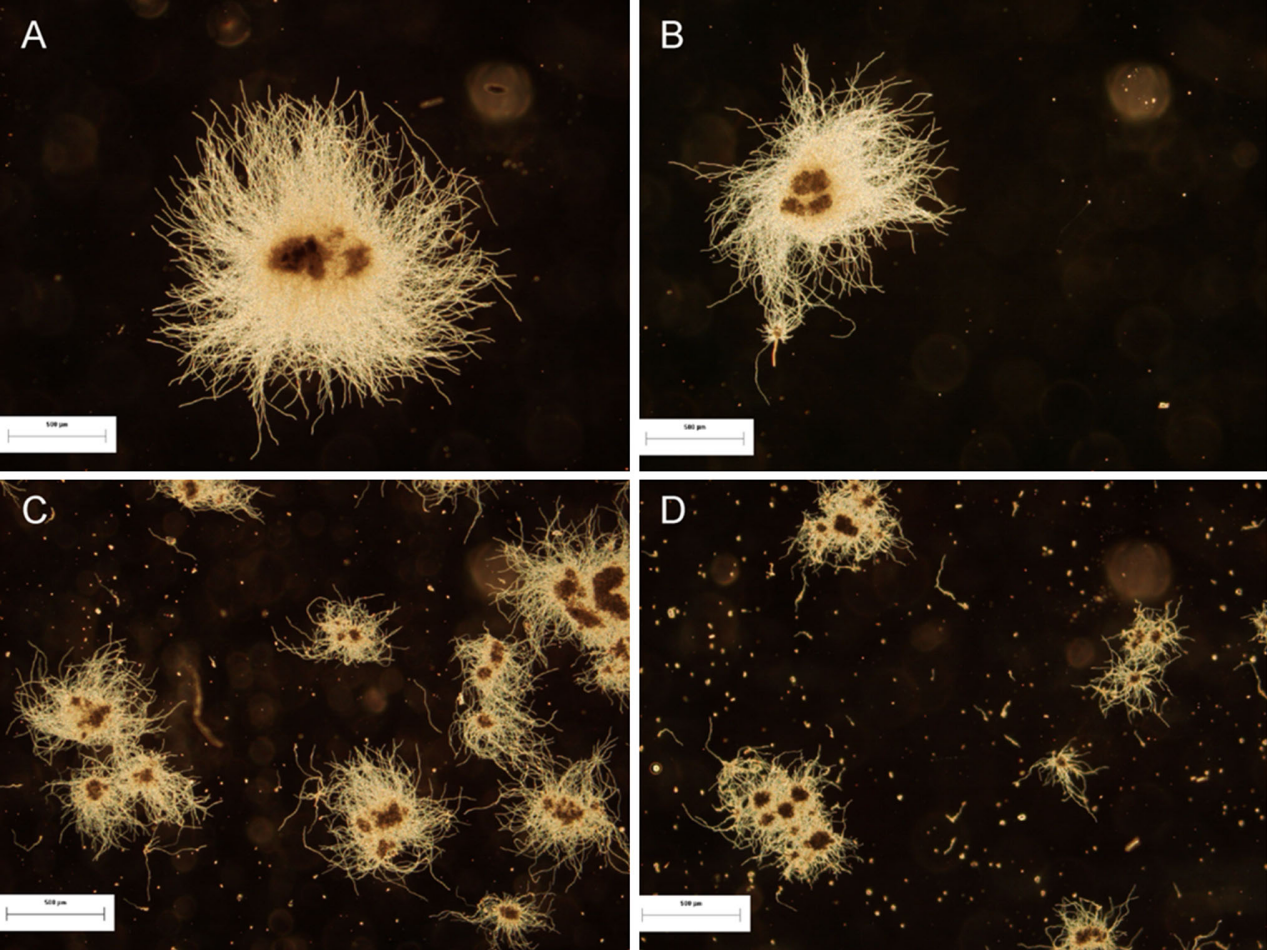
Images of pellets obtained at various talc microparticles concentrations (24 h of the preculture): **a** control, **b** 1 g l^−1^, **c** 6 g l^−1^ and **d** 15 g l^−1^; reproduced from Gonciarz and Bizukojc (2014) with permission granted from Wiley

For *A. terreus* ATCC 20542 progressively smaller pellets were obtained (Fig. 1) when increasing amounts of talc microparticles were added (Gonciarz and Bizukojc 2014).

In contrast, with the addition of titanate oxide the results were different. The pellet size was almost unchanged up to 5 g l^−1^ titanate but higher microparticle doses led to the formation of core–shell pellets (Driouch et al. 2012), which was actually a discovery of a new morphological form of *A. niger* SKAn 1015. The formed microparticle kernels provided solid support for the growth of biomass which during the process gradually covered the titanate cores with a thin layer of mycelium.

**Fig. 2:**
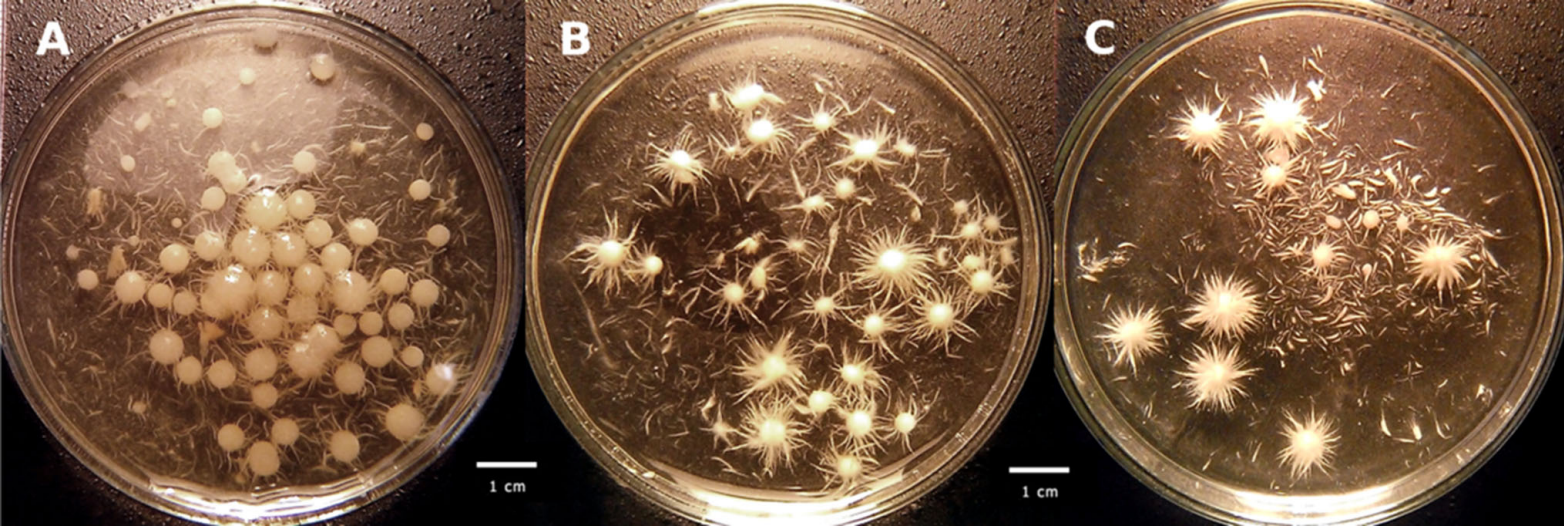
Influence of aluminum oxide microparticles added to the culture medium on *C. unicolor* morphology: **a** control run without microparticles, **b** 15 g l^−1^ and **c** 30 g l^−1^; *scale-bar size* 1 cm; from Antecka et al. (2016) distributed under the terms of the Creative Commons Attribution 4.0 International License

Coban et al. (2015a, b) reported that the addition of microparticles to the *A. ficcum* NRRL 3135 culture decreased average fungal pellet size and prevented bulk fungal growth. In shake flask cultivations the average fungal pellet radius decreased from 800 µm for control to 500 and 200 µm by increasing the particle concentration to 15 g l^−1^ of aluminum oxide and talc, respectively. The addition of talc microparticles to the preculture of *A. terreus* ATCC 20542 in a stirred tank bioreactor (Gonciarz et al. 2016) also promoted the formation of smaller pellets. Finally, rare shapes of pellets called star-shaped pellets (Fig. 2) were obtained during cultivation of the basidiomycetes strain *C. unicolor* in a culture with the addition of 15 g l^−1^ of aluminum oxide (Antecka et al. 2016). Surprisingly, another basidiomycetes strain, *P. sapidus*, changed its morphology in an all together different way after the addition of microparticles, and core–shell pellets, similar to the biomass of *A. niger* SKAn 1015 after the use of titanate microparticles, were achieved.

#### Change of oxygen transfer conditions and viscosity

One of the positive effects of MPEC is the improvement of oxygen transfer to the fungal cells due to formation of smaller pellets (Walisko et al. 2012; Driouch et al. 2010a; Gonciarz and Bizukojc 2014). Formation of mycelial clumps by filamentous fungi often causes difficulties with mixing and limitations in the diffusion of oxygen and nutrient supply to biomass (Gibbs et al. 2000), which reduce the capacity of microorganisms for growth and metabolite or enzyme production. By applying MPEC these difficulties can be significantly reduced or even eliminated as this method facilitates easier diffusion of oxygen to smaller fungal pellets. Gonciarz and Bizukojc (2014) proved that the positive effect of MPEC in lovastatin-producing *A. terreus* ATCC 20542 was associated with better aeration of fungal pellets, documented by the intrapellet oxygen concentration profiles. In another study by means of image analysis techniques, Driouch et al. (2010a), quantitatively determined and visualized metabolically active cells of the pellets (these ones to which nutrients and oxygen reached) by means of fluorescent probes. The result of this analysis was that only a thin layer on the outer surface of the pellets remained active and capable of fluorescent protein production. Notably, this active layer was enhanced and it was evidence for how efficient MPEC could be. The interaction between microparticles and fungal mycelium created a highly active biocatalyst with the dominant fraction of cells contributing to production (Driouch et al. 2010a).

Dispersed cells are decidedly better supplied with oxygen and nutrients than large cell pellets, where the inner core can consist of essentially inactive or even dead biomass (Driouch et al. 2012). On the other hand, the formation of free mycelium increases viscosity of the broth and decreases the effective oxygen interphase transport from gas phase to liquid phase. For instance, the viscosity of free mycelium of *A. niger* SKAn 1015 after addition of talc was 4-fold higher than that after addition of titanate when core–shell pellets were formed (Driouch et al. 2012). That is why the optimal culture condition and morphological form of the fungi should be simultaneously optimized to ensure high product secretion while at the same time maintaining low viscosity of the culture.

As mentioned earlier, fungal morphology influences the rheology of the cultivation medium. However, it has a significant impact not only on mixing but also mass transfer within the culture medium. Therefore, better mass transfer could also be the reason why MPEC works in this way. For instance, Coban et al. (2015a) observed that the addition of the same amount of talc makes fungal pellets smaller compared to those with aluminum oxide. These authors suggested that it was caused by the differences in mean particle sizes and shapes of these two different microparticles. Talc microparticles had a smaller diameter than those of aluminum oxide and provided better mass transfer in the culture medium. Additionally, one should take into account that talc is not completely inert (Etschmann et al. 2015). However, there is still no evidence whether more intensive biomass growth was caused directly by microparticles or due to microelements released from the microparticles.

#### Change of substrate consumption rates

Another positive effect of MPEC is the enhancement of uptake of carbon sources. It was shown by Gonciarz and Bizukojc (2014) that the presence of talc microparticles increased lactose uptake rate by lovastatin-producing *A. terreus* ATCC 20542. Substrate consumption was the slowest in the control run, and the fastest lactose uptake for talc concentration of 15 g l^−1^ was observed within the first 24 h of the cultivation. On the other hand, Etschmann et al. (2015) reported that for *T. atroviride* IMI 206040 cultivation in the control run about 78 % of the initial glucose was consumed, while in the cultures with microparticles it changed from 36 to 89 % dependent on the microparticles used. So, from their results, no correlation between glucose consumption and pellet size could be established.

#### Change of enzymes or metabolites titer

In most cases the researchers tried to establish an optimal concentration of microparticles that could increase fungal productivity. Kaup et al. (2008) obtained about 10-fold enhancement of chloroperoxidase production by *C. fumago* DSM 1256 after addition of talc with a concentration of 10 g l^−1^. Moreover, they noticed that the specific productivity of fungal biomass was also significantly enhanced. Also for organisms of the genus *Aspergilli* MPEC led to the increase of product formation: 4-fold higher of α-glucoamylase and β-fructofuranosidase secretion by *A. niger* (Driouch et al. 2010a), a 3.5-fold higher lovastatin titer produced by *A. terreus* ATCC 20542 (Gonciarz et al. 2016) or a 3-fold increase of phytase activity by *A. ficuum* NRRL 3135 (Coban et al. 2015a). The latest study also proved the positive effect of microparticles for basidiomycetes strains (Antecka et al. 2016). Despite the fact that fungal morphology for *C. unicolor* and *P. sapidus* were completely different, in both cases laccase activity was increased, 3.5 and 2-fold, respectively. Table 1 brings together results of MPEC showing positive effects when applied to filamentous fungi cultivation.

**Table 1:** Positive effects of MPEC methods on fungal biomass and productivity

Organism	Bioproduct	Microparticle optimal concentration (g l^−1^)	Biomass morphology	Effect (productivity increase)	References
*Caldariomyces fumago* DSM 1256	Chloroperoxi-dase	Talc (10)	Loosely packed mycelia with single hyphae	10-fold	Kaup et al. (2008)
		Aluminum oxide (15)		6-fold	Kaup et al. (2008)
*A. niger* AB1.13	α-glucoamylase	Talc (10)	Up to free mycelium	4-fold	Driouch et al. (2010a)
*A. niger* SKAn 1015	β-fructo-furanosidase	Talc (5)	Freely dispersed mycelium	10-fold	Driouch et al. (2010b)
*A. niger* SKAn 1015	β-fructo-furanosidase	Titanium silicate oxide (25)	Core–shell pellets	3.7-fold	Driouch et al. (2012)
*A. niger* ANip7-MCS-gfp2	α-glucoamylase	Titanium silicate oxide (25)	Core-shell pellets	9.5-fold	Driouch et al. (2012)
*A. terreus* ATCC 20542	Lovastatin	Talc (12)	Smaller pellets	1.6-fold	Gonciarz and Bizukojc (2014)
*Trichoderma atroviride* IMI 206040	6-pentyl-α-pyrone	Iron oxide (II,III) (20)	Pellets of various sizes	2-fold	Etschmann et al. (2015)
*A. niger* DSM 821	2-phenylethanol	Talc (20)	Homogeneous cultures	1.3-fold	Etschmann et al. (2015)
*A. ficuum* NRRL 3135	Phytase	Talc (15)	Smaller pellets	3-fold	Coban et al. (2015a)
		Aluminum oxide (15)		2-fold	Coban et al. (2015a)
*A. terreus* ATCC 20542	Lovastatin	Talc (12)	Smaller pellets	3.5-fold	Gonciarz et al. (2016)
*R. oryzae* NRRL 395	Lactic acid	Talc (10)	Smaller pellets	4-fold	Coban and Demirci (2016)
		Aluminum oxide (15)		2.3-fold	Coban and Demirci (2016)
*C. nicolor* (Bull. Ex Fr) Murr. 137	Laccase	Aluminum oxide (15)	Star-shaped pellets	3.5-fold	Antecka et al. (2016)
*P. sapidus* DSM 8266	Laccase	Aluminum oxide (15)	Core–shell pellets	2-fold	Antecka et al. (2016)
*A. sojae* AsT1	β-mannanase	Talc (5)	Smaller pellets	1.8-fold	Yatmaz et al. (2016)
		Aluminum oxide (1)		2.5-fold	Yatmaz et al. (2016)

When comparing the increase of product formation, it is seen that some fungi responded better to the particle addition than others. Etschmann et al. (2015) who studied above 15 various microparticles claimed that for *T. atroviride* IMI 206040 it was not possible to establish the correlation between pellet size and 6-pentyl-α-pyrone concentration. But, in the same study, they were able to choose the group of particles which enhanced 6-pentyl-α-pyrone formation. The highest concentration of 6-pentyl-α-pyrone was achieved after addition of iron oxide (II, III) or aluminum titanate, which caused about 2-fold increase of 6-pentyl-α-pyrone titer compared to unsupplemented control. Interestingly, for 2-phenylethanol production by *A. niger* DSM 821 the best result was obtained with the addition of 40 µm talc microparticles.

To sum up, it must be clearly stated that it is difficult to establish any general rules between filamentous fungi, microparticles used and the resulting fungal productivities. Each fungal species, microparticles and product should be analyzed individually in each given culture condition (Etschmann et al. 2015).

### How do microparticles act?

Presently, it is still unclear why the metabolites or enzymes displayed increased productivity in MPEC than in control cultivations. In the majority of works cited in this review not much was written about the mechanism of the action of microparticles. The question remains as to how the microparticles change the morphology of fungi and, as a consequence, increase productivity of strains. Only Driouch et al. (2010a) presented a short description of the interactions between microparticles and fungal cells following microscopic observations. They monitored the morphological development of *A. niger* at various time points during the initial stages of cultivation. In the control culture large agglomerates typically observed in submerged fungal cultures were maintained and then transformed into large solid pellets. When talc microparticles were added, the initially present large spore agglomerates disappeared, and during germination only individual spores were observed, resulting in a loose mycelium. However, when the microparticles were added at later stages of cultivation the morphology of *A. niger* was not affected. This indicates that the microparticles disturbed the initial phase of spore aggregation and was the reason for the change in morphology. Therefore, finding the moment of cultivation time to add the microparticles was crucial. It was proven that in order to induce the desired morphological changes of the growing mycelium microparticles must be added together with spores when the agglomerates are just beginning to form. According to the present knowledge, one can conclude that the action of microparticles relies the surface interaction between spores and microparticles and it is rather a kind of spatial and mechanical effect (assuming fully inert microparticles). Nevertheless, further physiological effects were induced as the microparticles promoted the formation of smaller pellets and facilitated access of oxygen to the mycelium. As a result, more efficient consumption of substrates and increase of fungal productivity were often observed (Driouch et al. 2010a, b; Gonciarz and Bizukojc 2014; Coban et al. 2015a).

## Changes of medium osmolality

Osmolality of the solution is defined as the number of moles of osmotically active substances per 1 kg of solvent. In microbiology this feature of the solution or cultivation medium is often referred to as water activity. Osmolality of the media strongly influences microbial growth. If it is too high, growth may completely cease. Typical osmolalities of cultivation media are between 0.28 and 0.32 osmol kg^−1^. Previously, it was observed that osmotic stress may induce the excretion of enzymes (Bobowicz-Lassociska and Grajek 1995; Fiedurek 1998). Common substances that can be used to cause osmotic stress are sodium and potassium chlorides, which are physiologically inert salts. Osmotic stress has rarely been proposed to control fungal morphology. In fact, Wucherpfennig et al. (2011) were the only team to do this, they proved that changing of osmolality can be the tool of morphological engineering. However, because osmolality is not as metabolism neutral as MPEC, in their study they had to distinguish the metabolic effect of sodium chloride from the morphological one. Wucherpfennig et al. (2011) studied two strains, previously studied in MPEC, *A. niger* SKAn1015 (β-fructofuranosidase producer) and AB1.13 (α-glucoamylase producer). They used a 3-l working volume stirred-tank bioreactor. The basic medium for *A. niger* SKAn1015 had an osmolality of 0.4 osmol kg^−1^ and the study was made in the range of 0.4–4.9 osmol kg^−1^. The maximum of fructofuranosidase activity (220 U ml^−1^) was found between 2.6 and 3.2 osmol kg^−1^. In the case of glucoamylase production by *A. niger* AB1.13 no optimum osmolality was found. Enzymatic activity achieved 22 U ml^−1^ with increasing osmolality from 0.2 osmol kg^−1^ (basic medium) to 2.4 osmol kg^−1^.

Osmolality decreased the size of fungal agglomerates. For *A niger* SKAn1015 mean projected area of pellets decreased from 1.5 × 10^6^ µm^2^ for 0.4 osmol kg^−1^ to below 2.5 × 10^5^ µm^2^ for 4.2 osmol kg^−1^. A further increase of sodium chloride led to the formation of dispersed mycelium. Furthermore, osmolality changed the size of pellets, which were not ideally spherical. In order to prove that changes in pellet morphology, not only osmotic stress, resulted in better production of enzymes (18-fold increase of β-fructofuranosidase activity compared to control), they proposed the original dimensionless morphology number:

$$Morphology number = \frac{2 \cdot \sqrt A \cdot S}{\sqrt \pi \cdot D \cdot E}$$

where A—projected area of the pellet (µm^2^), S—solidity of the pellet, D—maximum diameter of the pellet (µm), E—aspect ratio (length to width ratio).

Morphology number for ideally spherical pellets is equal to 1, while for the dispersed mycelium is equal to 0. The values in-between correlate to the variety of shapes and sizes of the pellets. Owing to this number, they could clearly show that the enzymatic specific activity (enzyme productivity) decreased exponentially with the morphology number. Dispersed mycelium proved to be more efficient. Exhibiting an inverse relationship, as pellets grew denser and more spherical (higher morphology number) less enzymes were produced.

Wucherpfennig et al. (2011) found that the increase of osmolality acted on fungal growth and subsequently fungal morphology in two ways. Firstly, it caused delayed initiation of growth, increasing the duration of lag-phase. Secondly, the duration of spore germination phase was also longer. These factors contributed to the formation of smaller pellets or dispersed mycelium. This strong effect of osmolality was also confirmed by the specific growth rate of biomass, which actually decreased by one magnitude from 0.16 to 0.02 h^−1^ when osmolality was changed from 0.4 to 4.9 osmol kg^−1^. Despite the slower evolution of the culture, Wucherpfennig et al. (2011) claimed that osmolality is a cheap and reliable approach to increase the productivity of industrial processes. Because of the predictable behavior of fungal morphology under osmotic stress, better control of the culture is possible.

## Summary and future studies

MPEC and changing osmolality are modern methods, which open new possibilities for tailor-made morphology design of filamentous fungi. According to the data in available literature, it seems to be especially useful for the fungi which have an agglomerative character of pellet formation from asexual spores. However, a full understanding of the mechanism of action of microparticles requires further investigation. It is known that the method works by decreasing the diameter of macropellets, ultimately changing the morphology into dispersed hyphae; however, there is presently a lack of any quantitative data describing this phenomenon. MPEC is to a small extent dependent upon the chemical particle composition. In the literature there are some examples of correlations between morphology and fungal productivity, additionally, the positive effects of MPEC seem to be strain and particle size dependent and require individual investigations in each case. Therefore, future studies in this field must be conducted, especially the study of species other than those belonging to the genus *Aspergillus*, whose growth with regard to morphology (mainly the mechanism of pellet formation) is different.

The successful use of osmolality changes for controlling fungal morphology still requires further research, as only one paper (Wucherpfennig et al. 2011) was devoted to it. Nevertheless, its authors introduced a valuable tool (morphology number) to study the control of fungal cultivations with the application of morphological engineering techniques.

Lastly, from an industrial point of view, it is important that the concept of morphological engineering was successfully transferred from flasks into bioreactor (Driouch et al. 2010a, b, 2012; Coban et al. 2015b; Gonciarz et al. 2016). The relatively low costs of the method (inexpensive microparticles added in low amounts and sodium chloride) is of great relevance for future large scale applications.
